# Supplementary material for: Photothermal catalytic transfer hydrogenolysis of protolignin
Source: Nat Commun. 2024 Nov 23;15:10176. doi: 10.1038/s41467-024-54664-6 (PMC11585588; doi:10.1038/s41467-024-54664-6)
Supplement: Supplementary file 1 — Supplementary Information [file 41467_2024_54664_MOESM1_ESM.pdf]

Supplementary Information

Photothermal Catalytic Transfer Hydrogenolysis of Protolignin

Hongji Li[1]*, Xiaotong Sun[1], Ting Li[2], Zhitong Zhao[3], Hui Wang[1], Xiaomei Yang[1], Chaofeng Zhang[2]*, and Feng Wang[4,5]*

[1] College of Chemistry, Zhengzhou University, 100 Science Avenue, Zhengzhou, 450001, China. Email: hongjili@zzu.edu.cn

[2] Jiangsu Co-Innovation Center of Efficient Processing and Utilization of Forest Resources, College of Light Industry and Food Engineering, Nanjing Forestry University, 159 LongPan Road, Nanjing 210037, China. Email: zhangchaofeng@njfu.edu.cn

[3] College of Chemical Engineering and Technology, Taiyuan University of Technology, Taiyuan 030024, China.

[4] State Key Laboratory of Catalysis, Dalian National Laboratory for Clean Energy, Dalian Institute of Chemical Physics, Chinese Academy of Sciences, 457 Zhongshan Road, Dalian 116023, China. Email: wangfeng@dicp.ac.cn

[5] University of Chinese Academy of Sciences, Beijing 100049, China

Contents

Supplementary Methods	Page S4
Table S1 | Comparison of photothermal catalytic transfer hydrogenolysis of protolignin with previous representative works on thermal-catalytic and photocatalytic transfer hydrogenolysis of lignin	Page S5
Fig. S1 | The photothermal catalytic transformation of lignin model in 1-propanol using Pt/TiO_2	Page S8
Fig. S2 | The photothermal catalytic transformation of lignin model in 1-butanol using Pt/TiO_2	Page S9
Fig. S3 | The GC data of TiO_2 catalyzed the transformation of model **1** under photothermal conditions	Page S10
Fig. S4 | The GC data of 3%-Pd/TiO_2 catalyzed the transformation of model 1 under photothermal conditions	Page S11
Fig. S5 | The XRD pattern of prepared 3%-Pd/TiO_2	Page S12
Table S2 | The BET analysis of photocatalysts	Page S13
Fig. S6 | The SEM and TEM images of catalysts	Page S14
Fig. S7 | The TEM-EDS mapping images of 3%-Pd/TiO_2	Page S15
Fig. S8 | The photo-responsive properties of photocatalysts	Page S16
Fig. S9 | The photothermal catalytic transfer hydrogenolysis of lignin model in different alcohols	Page S17
Fig. S10 | The proposed elementary steps involved in the transfer hydrogenolysis	Page S18
Fig. S11 | The kinetic data used for determining the initial rate of model **1**	Page S18
Table S3 | The effect of the light source on photothermal catalytic transfer hydrogenolysis of lignin model	Page S19
Table S4 | The photothermal catalytic test of Pd-loaded catalysts under UV and visible light irradiation	Page S20
Table S5 | The photothermal catalytic test of Ni-loaded catalysts	Page S21
Fig. S12 | The photocurrent test of 3%-Pd/TiO_2 under 370 nm LED irradiation at different temperature	Page S22
Fig. S13 | The GC-MS analysis of depolymerized products from hydrogenolysis of birch sawdust	Pages S23
Fig. S14 | The mass spectra of lignin monomers from hydrogenolysis of birch sawdust	Page S25
Table S6 | The analysis of small products after transfer hydrogenolysis of birch sawdust	Page S26

Table S7 | The lignin content in various biomass substrates	Page S27
Fig. S15 | Multiple roles of HCl during photothermal catalytic transformation of lignocellulose	Page S28
Table S8 | Composition of wheat straw used for TEA and LCA analysis	Page S29
Fig. S16 | Process flow model of two scenarios	Page S31
Table S9 | Key stream for process flow model	Page S32
Table S10 | Key parameters for techno-economic analysis	Page S35
Fig. S17 | The detailed breakdown of total production cost (TPC) of 4-propylguaiacol in Scenario 2	Page S36
Fig. S18 | System boundary of this work	Page S37
Fig. S19 | The detailed breakdown of life cycle GHG emissions of 4-propylguaiacol in Scenario 2	Page S38
NMR data and spectra of lignin models	Page S39
Supplementary References	Page S43

Supplementary Methods

The organic chemicals, metal salts, and metal oxide nanoparticles were of analytical grade and purchased from commercial suppliers without further purifications.

The lignin β-O-4 diol model was prepared according to our previous works [1,2]. The ethylidene acetal protected β-O-4 model was obtained from the Pt/TiO_2 photothermal catalyzed conversion of the β-O-4 diol model at 140 °C and purified via column chromatography. The 2-benzyl-3-(2-methoxyphenyl)propan-1-ol was obtained from the Pd/TiO_2 photocatalyzed conversion of β-O-4 diol model at 30 °C and purified via column chromatography.

Supplementary Discussion

Table S1 | Comparison of photothermal catalytic transfer hydrogenolysis of protolignin with previous representative works on thermal-catalytic and photocatalytic transfer hydrogenolysis of lignin

Entry	Substrate	Catalyst	Temperature (°C)	hυ	Solvent	Time (h)	Yield[a] (wt%)	Main products	Ref.
1	Birch	$Ni_{50}Pd_{50}$/SBA-15	245	-	2-PrOH /H_2O	4	37	4-Propylsyringol, 4-propenylsyringol, 4-propylguaiacol, 4-propenylguaiacol	3
2	Poplar	Pd/C	225	-	MeOH	3	28	Propyl and ethyl-substituted monomers	4
3	Poplar	Pd/C	225	-	Ethylene glycol	3	21	Propyl and ethyl-substituted monomers	5
4	Swedish birch	Pd/C	210	-	EtOH/H_2O	2	40	4-Propylsyringol, 4-propenylsyringol	6
5	Birch	Ni/C	200	-	MeOH	6	54	4-Propylsyringol, 4-propylguaiacol	7
6	Hemp Hurd	Pd/C+*p*-toluenesulfonic acid	200	-	MeOH/H_2O (HCOOH)	4	38	monophenols	8
7	Birch	Co-phen/C	200	-	EtOH/H_2O (5 equiv. HCOOH and 5 equiv. HCOONa)	4	34	4-Propylsyringol, 4-propenylsyringol, 4-propylguaiacol, 4-propenylguaiacol	9
8	Birch	Pt/C	190 (microwave)	-	MeOH/ H_2O	3	29	4-Propyl syringol, 4-propenyl syringol	10

9	Birch	Ru/C	190	-	Ethylene glycol (10 wt% choline chloride)	8	59	Propylphenol	11
10	Poplar	$Ru/C+H_2SO_4$	185-195	-	Ethylene glycol	6	27	4-Propylsyringol, 4-propylguaiacol	12
11	Poplar	Pd-PdO/TiO2	180	-	H_2O (STH)	6	40	monophenols	13
12	Bagasse	$Pd/AC+H_4SiW_{12}O_{40}$	170	-	2-PrOH	5	35	4-Ethylphenol, 4-ethylguaiacol	14
13	Birch	$Pt/NiAl_2O_4$	140	-	H_2O (hemicellulose as HD)	24	47	Propyl and ethyl-substituted monomers	15
14	Poplar	Rhodium terpyridine complexes	110	-	H_2O (STH)	12	17	Aromatic monomers	16
15	Rice-straw lignin	NiMo-MACS	340	-	Formic acid/EtOH	6	72	Oil	17
16	Technical lignin	-	300	-	EtOH	4	21	Phenolic monomers	18
17	Kraft lignin	Co/carbon nanotube	280	-	EtOH	0.5	66	Bio-oil	19
18	Organosolv poplar lignin	$Ni_{10}Cu_5/C$	270	-	EtOH/2-PrOH	4	63	Propyl and ethyl-substituted monomers	20
19	Cornstalk hydrolysis residue	Ru/AC	260	-	Ethyl acetate/H_2O	5	43	Aromatics	21
20	Acid-extracted birch lignin	$PtRe/TiO_2$	240	-	2-PrOH/ H_2O	12	19	Monophenols	22
21	Organosolv poplar lignin	ReO_x/AC	200	-	2-PrOH	8	11	Phenolic monomers	23
22	Organosolv birch lignin	Pd1Ni4/MIL-100 (Fe)	180	-	H_2O (STH)	6	17	Guaiacol, 4-methoxyacetophenone	24

23	Dioxasolv beech lignin	Ni/Al_2O_3-600	170	-	2-PrOH	12	13	Monomers	25
24	Birch	Thiol-capped ultrathin $ZnIn_2S_4$ microbelts	Ambient temperature	450 nm	CH_3CN/H_2O	8	29	Syringyl and guaiacyl -derived ketones	26
25	Birch	CdS quantum dots	Room temperature	420-780 nm	$MeOH/H_2O$	8	27	Syringyl and guaiacyl -derived ketones	27
26	Dioxanesolv poplar lignin	$ZnIn_2S_4$	42	455 nm	Acetone/2-PrOH	24	10	Monophenols	28
**27**	**Birch**	**$Pd/TiO_2/HCl$**	**140**	**370 nm**	**EtOH/dioxane**	**8**	**40**	**4-Propylsyringol, 4-propylguaiacol**	**This work**
**28**	**Birch**	**$Pd/TiO_2/HCl$**	**Focused sunlight**		**EtOH/dioxane**	**6**	**34**	**4-Propylsyringol, 4-propylguaiacol**	**This work**
Note: HD, hydrogen donor; STH, self-hydrogen transfer. (a) Aromatic monomers.									

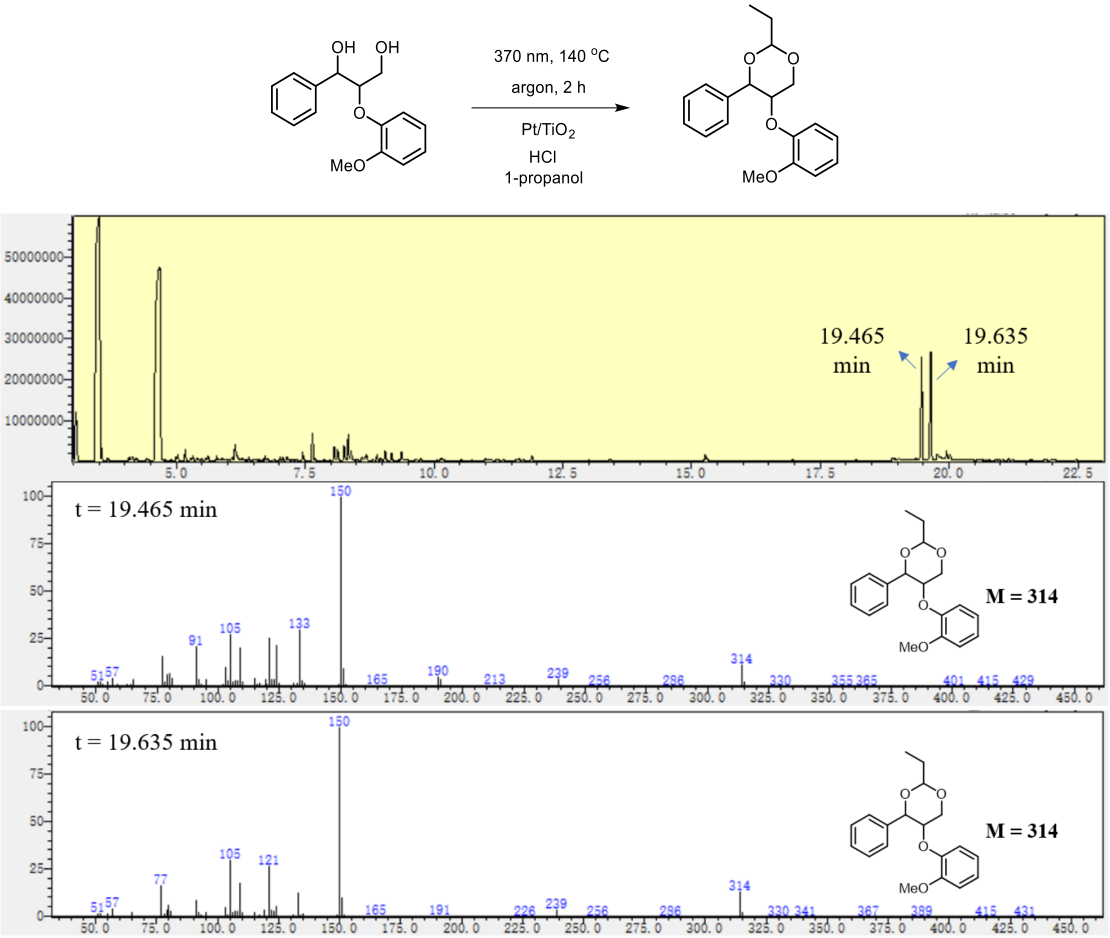

Fig. S1 | The photothermal catalytic transformation of lignin model in 1-propanol using Pt/TiO₂

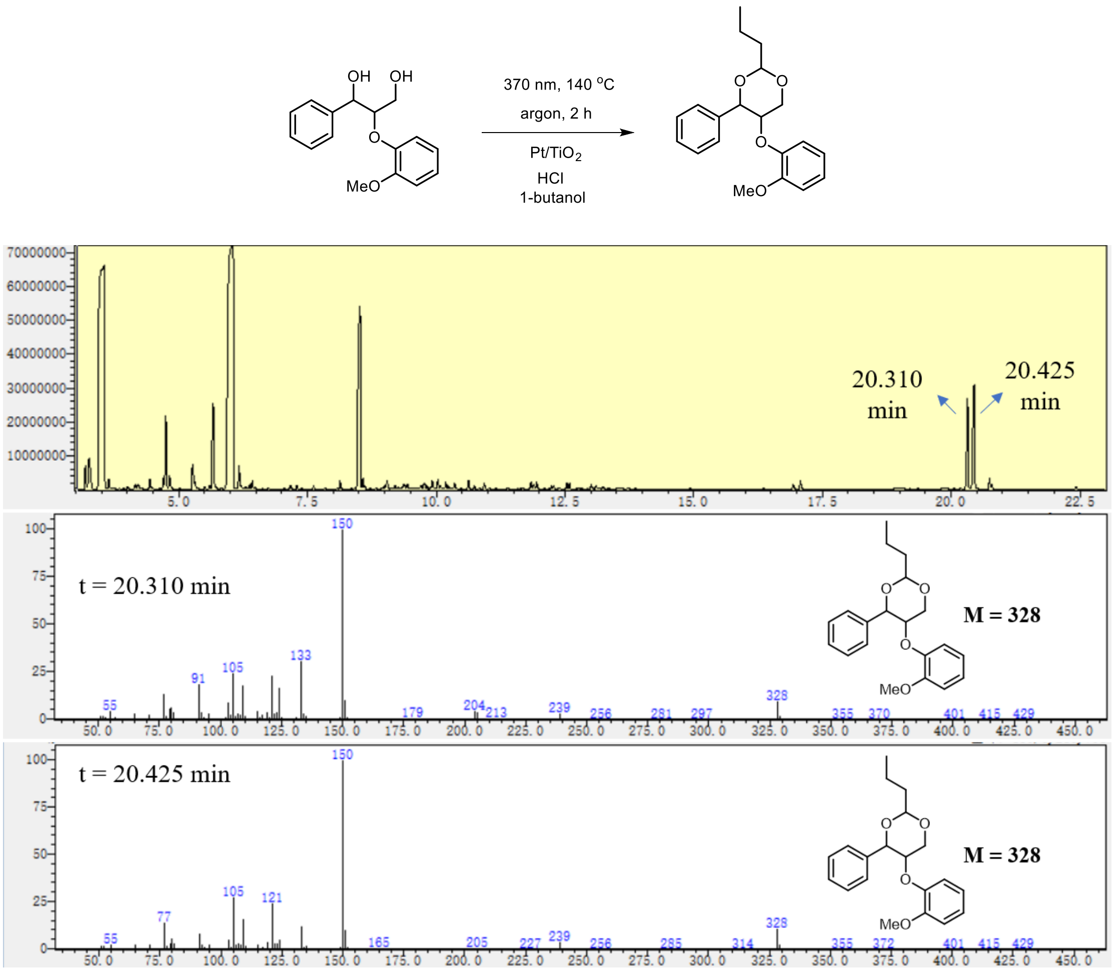

Fig. S2 | The photothermal catalytic transformation of lignin model in 1-butanol using Pt/TiO$_2$

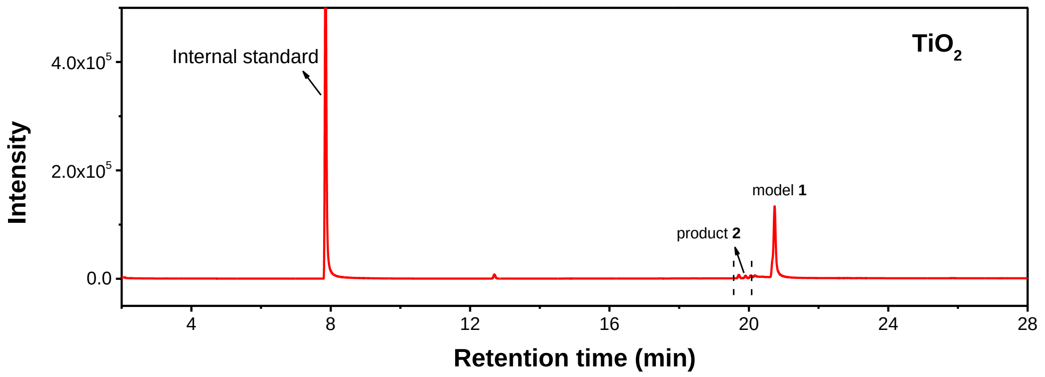

Fig. S3 | The GC data of TiO_2 catalyzed the transformation of model 1 under photothermal conditions

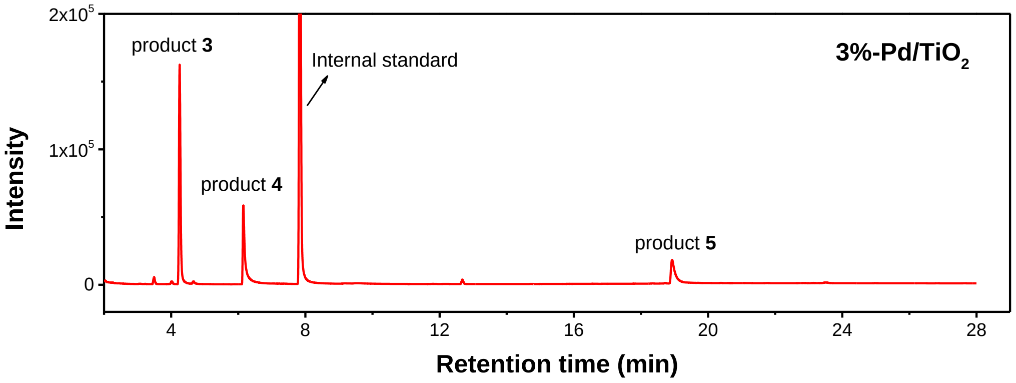

Fig. S4 | The GC data of 3%-Pd/TiO2 catalyzed transformation of model 1 under photothermal conditions

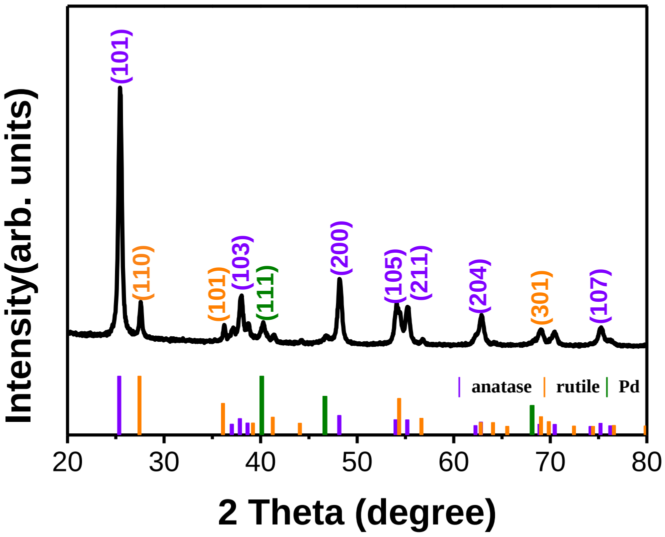

Fig. S5 | The XRD pattern of prepared 3%-Pd/TiO_2

Table S2 | The BET analysis of photocatalysts

Sample	BET surface area (m^2/g)
TiO_2	63.9
3%-Pd/TiO_2	47.7
6%-Pd/TiO_2	46.4

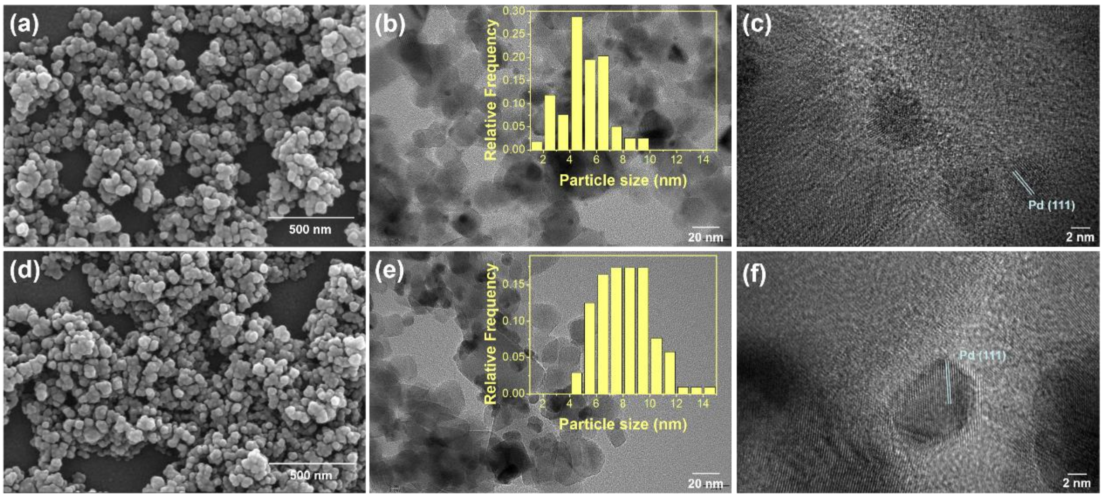

Fig. S6 | The SEM and TEM images of catalysts. The SEM (a) and TEM images (b, c) of 3%-Pd/TiO_2, and SEM (d) and TEM images (e, f) of 6%-Pd/TiO_2

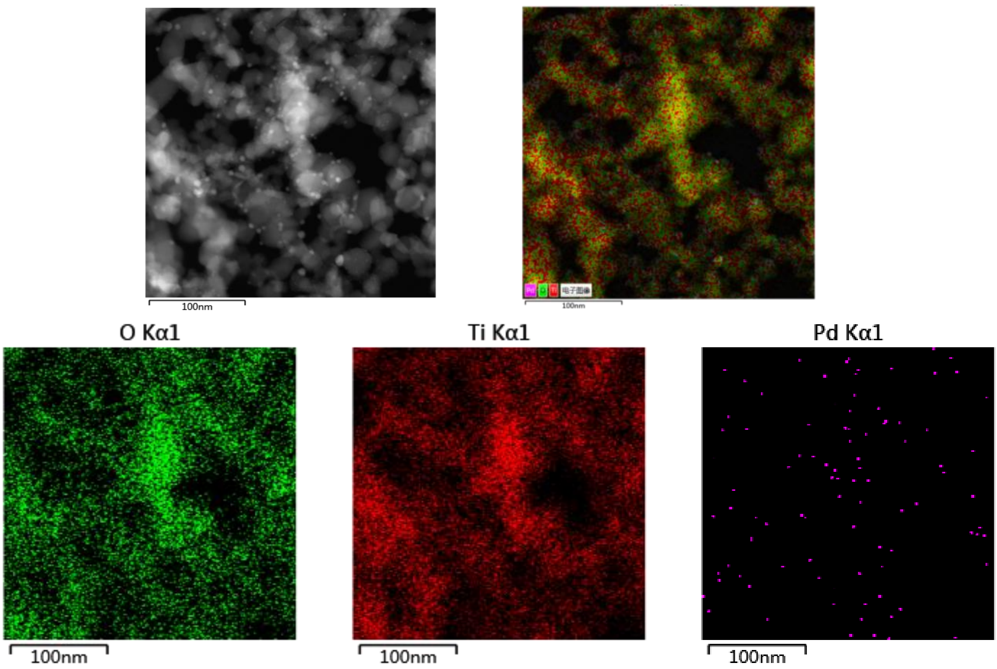

Fig. S7 | The TEM-EDS mapping images of 3%-Pd/TiO_2

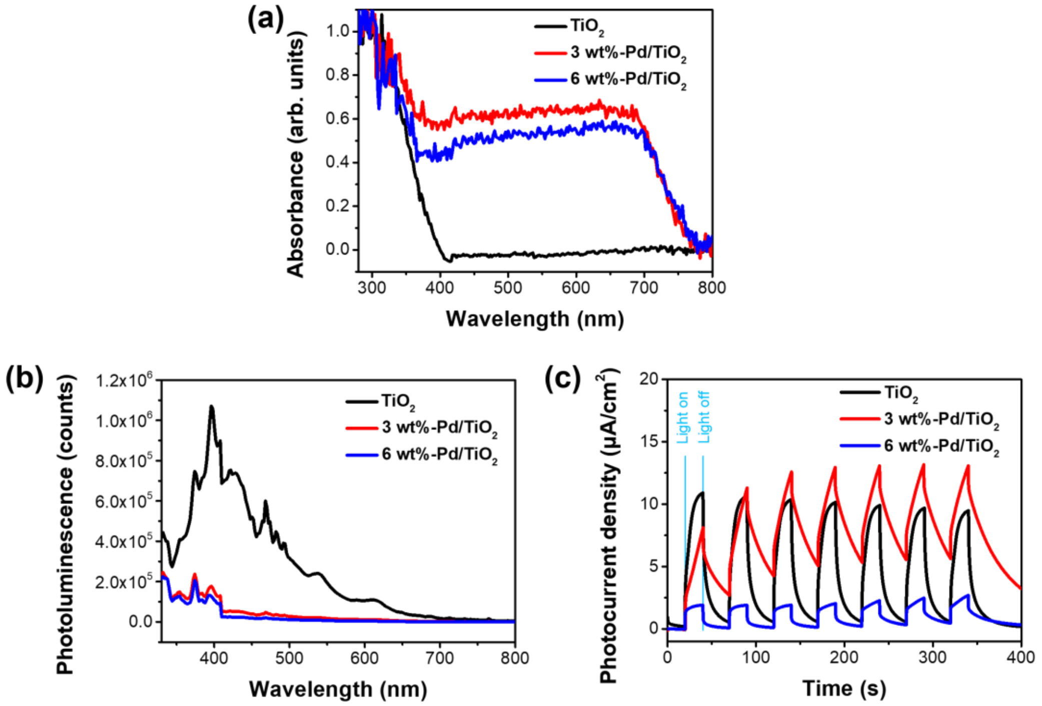

Fig. S8 | The photo-responsive properties of photocatalysts. (a) UV-vis absorption; (b) Fluorescence spectra (λ_{EX} = 300 nm); (c) Photocurrent test (under 370 nm LED irradiation).

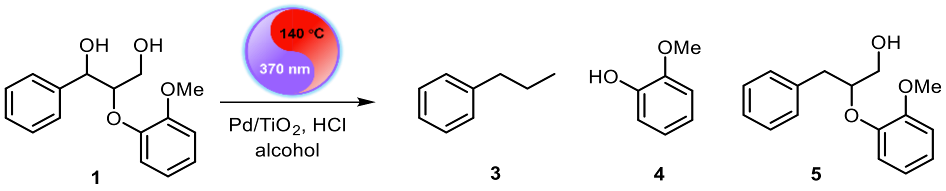

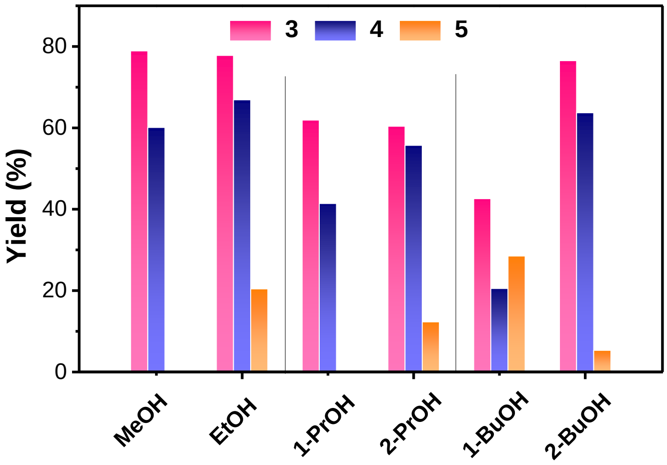

Fig. S9 | The photothermal catalytic transfer hydrogenolysis of lignin model in different alcohols

The apparent quantum efficiency (ζ) of transfer hydrogenation of the lignin model was calculated.

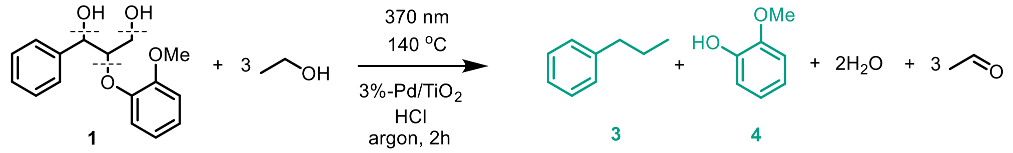

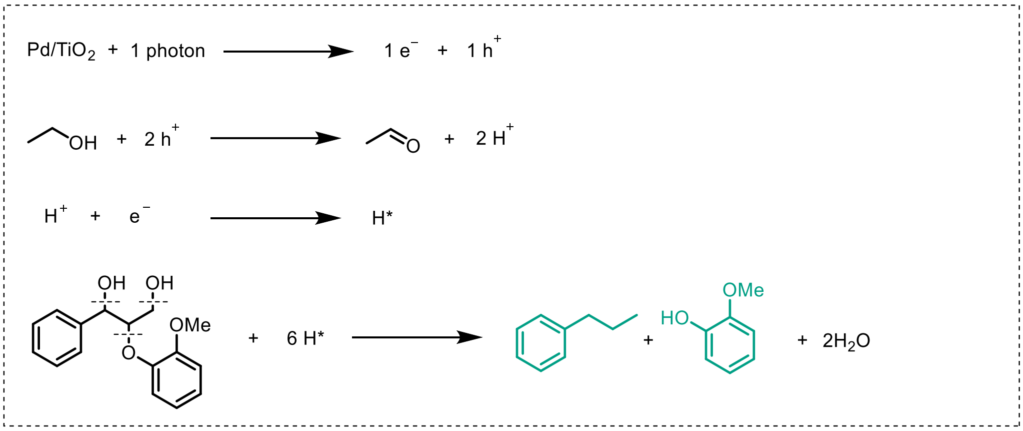

Fig. S10 | The proposed elementary steps involved in the transfer hydrogenolysis

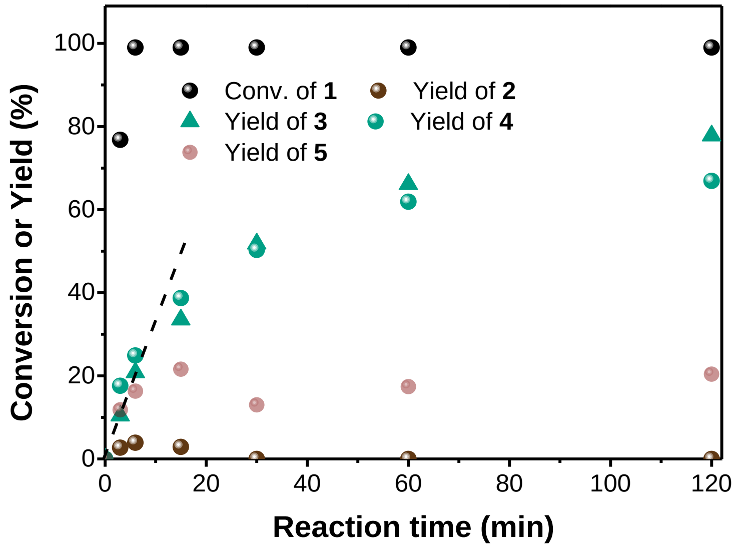

Fig. S11 | The kinetic data used for determining the initial rate of model 1

The transformation data at the initial stage (10 min) was used to calculate the kinetic data.

rate(H*) = rate(**3**) × 6 = n(**3**)/t × 6 = (10 mg)/(272 mg/mmol) × 0.208/(0.1 h) × 6 = 0.459 mmol/h

I_0(370 nm) = P/E(370 nm)/N_A = Avg. Intensity × Area × $\lambda/hc/N_A$ = (137 mW/cm^2)×(1.8 cm^2)/(6.626×10^{-34} J·s)/(299792458 m/s)×(370 ×10^{-9} m)/(6.022×10^{23} mol^{-1}) = 7.63×10^{-7} mol/s = 2.74 mmol/h

ζ = rate/I_0 × 100% = 16.7%

Table S3 | The effect of the light source on photothermal catalytic transfer hydrogenolysis of lignin model

Entry	Wavelength (nm)	Input power (W)	Yield of 3 (%)	Yield of 4 (%)	Yield of 5 (%)
1	370	0	0	0	0
2	370	10	56	18	22
3	370	20	62	45	16
**4**	**370**	**40**	**78**	**67**	**20**
5	427	40	0	0	0
6	456	40	0	0	0

Conditions: substrate **1** (10 mg), 3%-Pd/TiO_2 (10 mg), EtOH (1 mL), HCl (37%, 10 μL), Kessil LED (370/427/456 nm), 0-40 W, 140 °C, argon, 2 h.

Table S4 | The photothermal catalytic test of Pd-loaded catalysts under UV and visible light irradiation

Entry	Catalyst	Light wavelength (nm)	Yield of 2 (%)	Yield of 3 (%)	Yield of 4 (%)	Yield of 5 (%)
1	Pd/anatase	370	3	44	46	13
2	Pd/rutile	370	33	4	8	6
3	Pd/CeO_2	370	0	6	25	0
4	Pd/Nb_2O_5	370	7	5	17	0
5	Pd/ZnO	370	0	0	31	0
6	Pd/ZrO_2	370	0	0	0	0
7	Pd/ZrO_2	427	0	0	0	0
8	Pd/SiO_2	370	0	0	0	0
9	Pd/SiO_2	427	0	0	0	0
10	Pd/g-C_3N_4	370	12	42	45	0
11	Pd/g-C_3N_4	427	9	42	40	24

Conditions: substrate **1** (10 mg), catalyst (3 wt% Pd,10 mg), EtOH (1 mL), HCl (37%, 10 μL), Kessil LED (370/427 nm), 140 °C, argon, 2 h.

Table S5 | The photothermal catalytic test of Ni-loaded catalysts.

Entry	Catalyst	Yield of 2 (%)	Yield of 3 (%)	Yield of 4 (%)	Yield of 5 (%)
1	Ni/TiO_2-400	57	0	0	0
2	Ni/TiO_2-500	40	0	0	0
3	Ni/TiO_2-600	46	0	0	0

Conditions: substrate **1** (10 mg), catalyst (3 wt% Ni,10 mg), EtOH (1 mL), HCl (37%, 10 μL), Kessil LED (370 nm), 140 °C, argon, 2 h. The catalysts reduced with hydrogen under 400, 500, and 600 °C were denoted as Ni/TiO_2-400, Ni/TiO_2-500, and Ni/TiO_2-600, respectively.

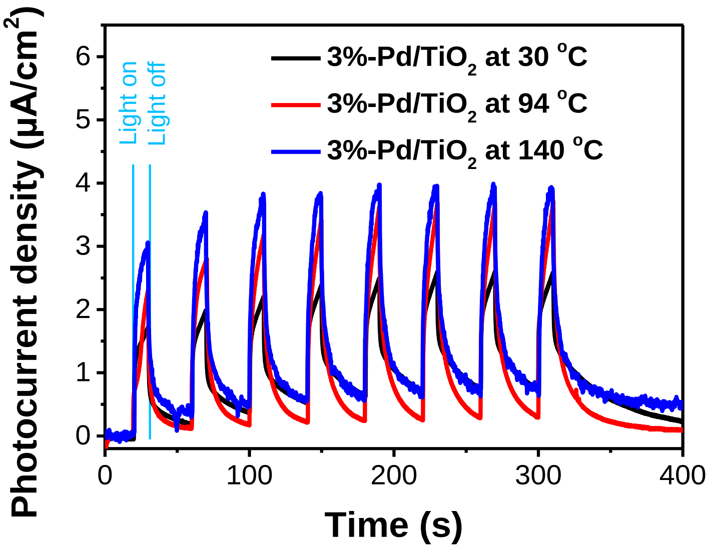

Fig. S12 | The photocurrent test of 3%-Pd/TiO_2 under 370 nm LED irradiation at different temperatures

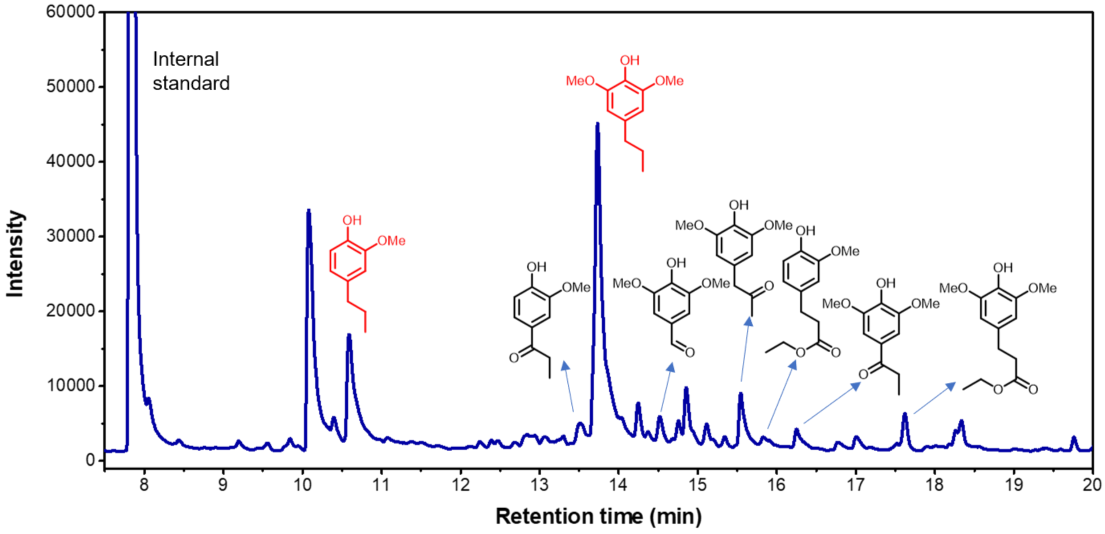

Fig. S13 | The GC-MS analysis of depolymerized products from hydrogenolysis of birch sawdust

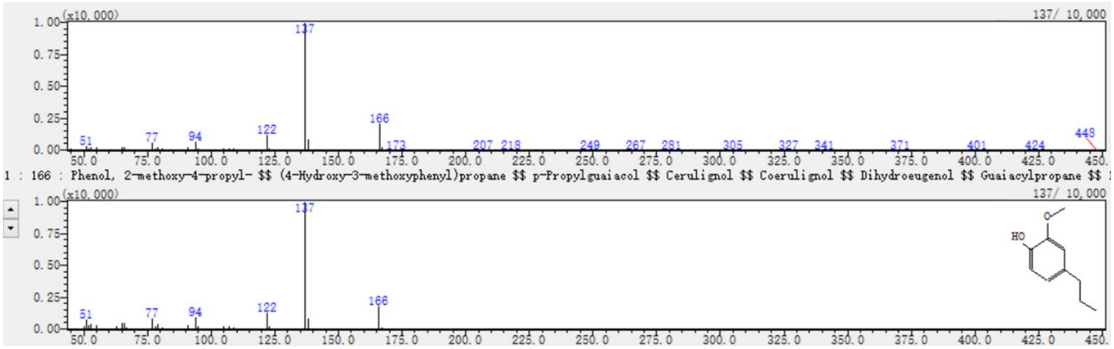
1 : 166 : Phenol, 2-methoxy-4-propyl- $$ (4-Hydroxy-3-methoxyphenyl)propane $$ p-Propylguaiacol $$ Cerulignol $$ Coerulignol $$ Dihydroeugenol $$ Guaiacylpropane $$
137/ 10, 000
HO

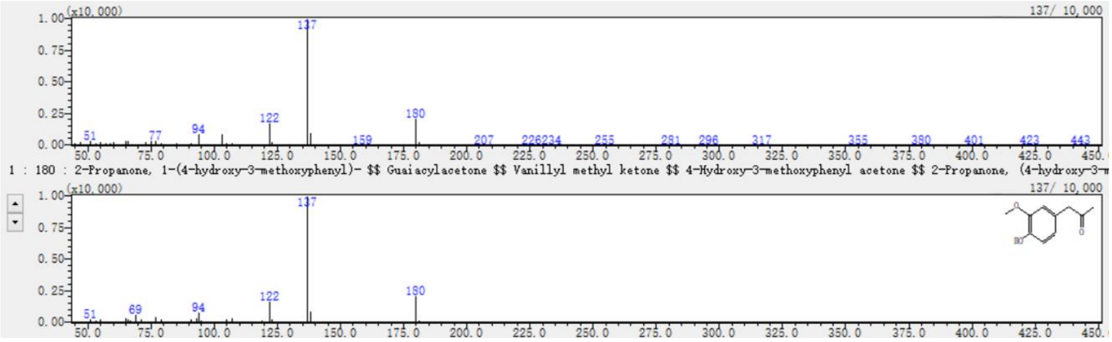
1 : 180 : 2-Propanone, 1-(4-hydroxy-3-methoxyphenyl)- $$ Guaiacylacetone $$ Vanillyl methyl ketone $$ 4-Hydroxy-3-methoxyphenyl acetone $$ 2-Propanone, (4-hydroxy-3-m
137/ 10, 000

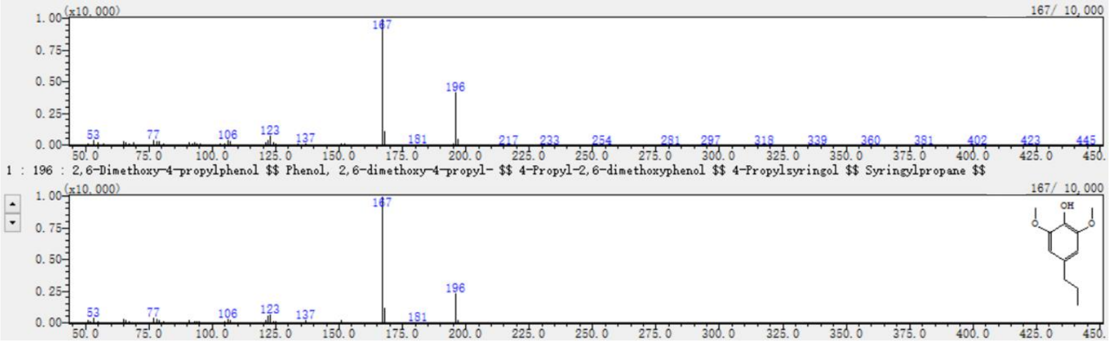
1 : 196 : 2,6-Dimethoxy-4-propylphenol $$ Phenol, 2,6-dimethoxy-4-propyl- $$ 4-Propyl-2,6-dimethoxyphenol $$ 4-Propylsyringol $$ Syringylpropane $$
167/ 10, 000
OH

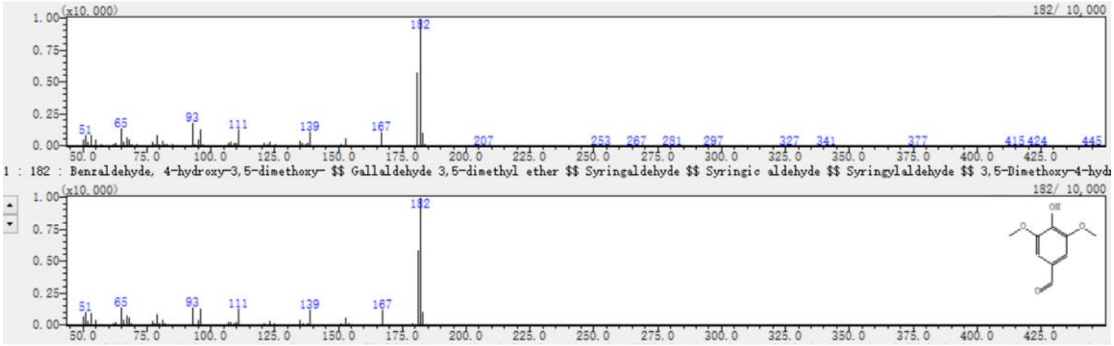
1 : 182 : Benzaldehyde, 4-hydroxy-3,5-dimethoxy- $$ Gallaldehyde 3,5-dimethyl ether $$ Syringaldehyde $$ Syringic aldehyde $$ Syringylaldehyde $$ 3,5-Dimethoxy-4-hydr
182/ 10, 000
OH

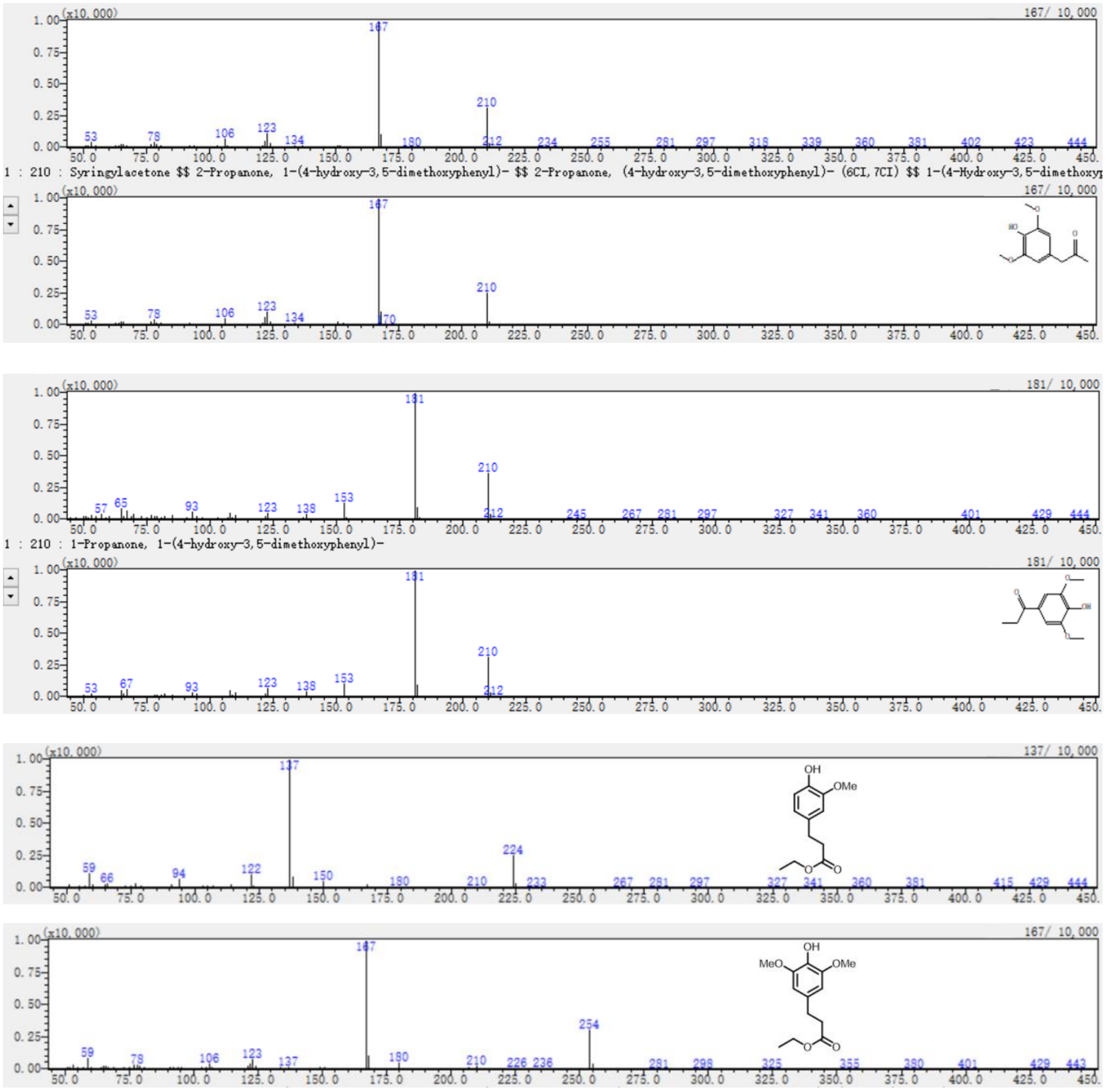

Fig. S14 | The mass spectra of lignin monomers from hydrogenolysis of birch sawdust

Table S6 | The analysis of small products after transfer hydrogenolysis of birch sawdust

Products		Reaction with HCl	Reaction with Pd/TiO_2 and HCl
Lignin products/wt%	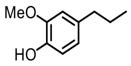	0	7
	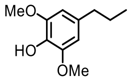	0	23
	others	0	10
Hemicellulose products/wt%	lactic acid	36	20
	xylose	22	10
	arabinose	27	10
	levulinic acid	1	2
	acetic acid	1	1
Residue-cellulose/wt%		66	59

Conditions: birch sawdust (60 mg), 3%-Pd/TiO_2 (0 or 10 mg), EtOH (1 mL), dioxane (0.6 mL), HCl (37%, 10 μL), Kessil LED (370 nm), 140 °C, argon atmosphere. The yields of lignin products were determined by GC. The yields of hemicellulose products were determined by HPLC. The quantification of residue cellulose was determined by the amount of glucose obtained from the further acid-catalyzed hydrolysis of birch solid residue after the photothermal reaction.

Table S7 | The lignin content in various biomass substrates

Biomass substrates	Birch	Pine	Platanus orientalis Linn.	Walnut shell	Reed straw	Wheat straw	Corn straw
Lignin （wt%）	19	22	22	19	21	13	7

Note: The lignin contents were determined using the NREL method.

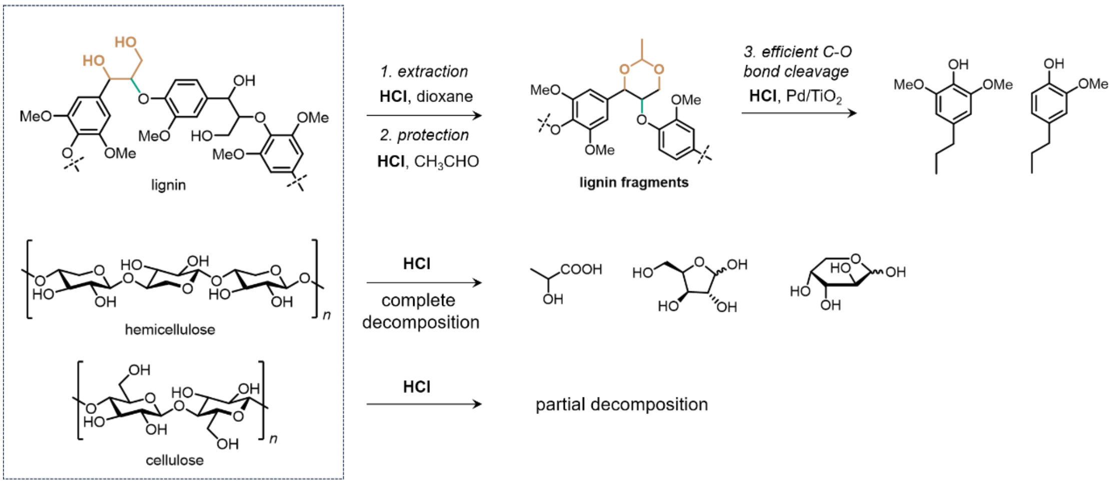

Fig. S15 | Multiple roles of HCl during photothermal catalytic transformation of lignocellulose

Process design details (TEA and LCA)

The process design is divided into two major sections: solar-driven photothermal reaction of protolignin, and cellulose hydrolysis and photocatalyst recovery. The front process is further split into feedstock pretreatment, photothermal reaction, gas and consumable recovery, residue separation, and 4-propylguaiacol purification units, while cellulose hydrolysis and photocatalyst recovery include unreacted cellulose hydrolysis and photocatalyst recovery units. Process design details are depicted in the following section.

The solar-driven photothermal reaction of protolignin

The feedstock is wheat straw that consists of cellulose, hemicellulose, lignin, ash, and water, as shown in Table S1. The milled wheat straw, together with process water, photocatalyst, hydrochloric acid, ethanol, dioxane, and argon flow, was heated for 50 °C and then fed into the photoreactor, where the catalytic reaction occurs under solar-driven photothermal conditions for a certain time. The hemicellulose and lignin were broken free from wheat straw and further converted into 4-propylguaiacol, monomers, lactic acid, arabinose, xylose, acetic acid, levulinic acid, polysaccharide. The cellulose and ash were sunk into slurry. Thus, the outlet mixture consists of the products derived from hemicellulose and lignin, slurry from cellulose and ash, together with catalyst, solvent, and argon atmosphere.

The separation sequence was arranged to obtain 4-propylguaiacol with a purity of 99.5wt% as well as co-products recovery. At first, the mixture products were fed into the gas-liquid separator and filter-press to remove gas- and solid-phase products. The gas was mainly argon used to be recycled as feedstock, while the solid contains cellulose, ash, and photocatalyst that requires further separation in the unit of cellulose hydrolysis and photocatalyst recovery. The residual mixture was fed into a flash tank at 200 °C to evaporate liquids with low boiling points, such as ethanol, dioxane, and water, which are solvents of photoreaction and recycled. The rest of the liquids with a high boiling point were fed into a decanter to remove hydrophilic components through solubility in water, and then the 4-propylguaiacol and some dimers were obtained.

Finally, a distillation column was assigned to recover the 4-propylguaiacol with designated purity.

Table S8 | Composition of wheat straw used for TEA and LCA analysis

Component	Content	Component in simulation model
Cellulose	33.0 wt%	Cellulose
Hemicellulose	23.2 wt%	Dl-Xylose
Lignin	13.0 wt%	Vanillin
Soluble components	18.8 wt%	Assumed to be glucose
Ash	12.0 wt%	Unconventional component

Slurry concentration and photocatalyst recovery

The slurry obtained from filter contains cellulose, ash, and photocatalyst, in which photocatalyst is required to recover due to its high cost. In addition, cellulose hydrolysis can produce costly glucose but suffers from a complex process, thus two scenarios with or not a cellulose hydrolysis process are arranged to identify higher economic performance and lower carbon emissions. This part is to reach cellulose hydrolysis and photocatalyst recovery.

Scenario 1: The cellulose hydrolysis occurs under 130 °C with the participation of sulfuric acid. Specifically, slurry derived from the filter was successively fed into a washer and digester. The washer was full of sulfuric acid at 45 °C while the digester was heated to 130 °C. During this process, nearly all cellulose was broken down into glucose. Then the mixture delivered from the digester cooled and removed the aqueous solution, such as H_2SO_4, glucose, etc. The rest entered the incinerator to burn solid organics and recover the photocatalyst, meanwhile, the steam generated supplied for whole heat network.

Scenario 2: Slurry derived from the filter was delivered into the incinerator to burn solid organics to recover photocatalyst and generate steam.

According to previous work and the above parameters, the overall process design

was simulated by Aspen plus to estimate detailed material and energy flow. The assumed input mass of wheat straw is 100,000 metric tons per annual (t/a). The output amount of 4-propylguaiacol will be 2800 t/a, which is a receivability scale. The process flow model is shown in Fig. S16. The components of the key stream are shown in Table S9. Note that the steam derived from the incinerator is sufficient for the whole plant in Scenario 2.

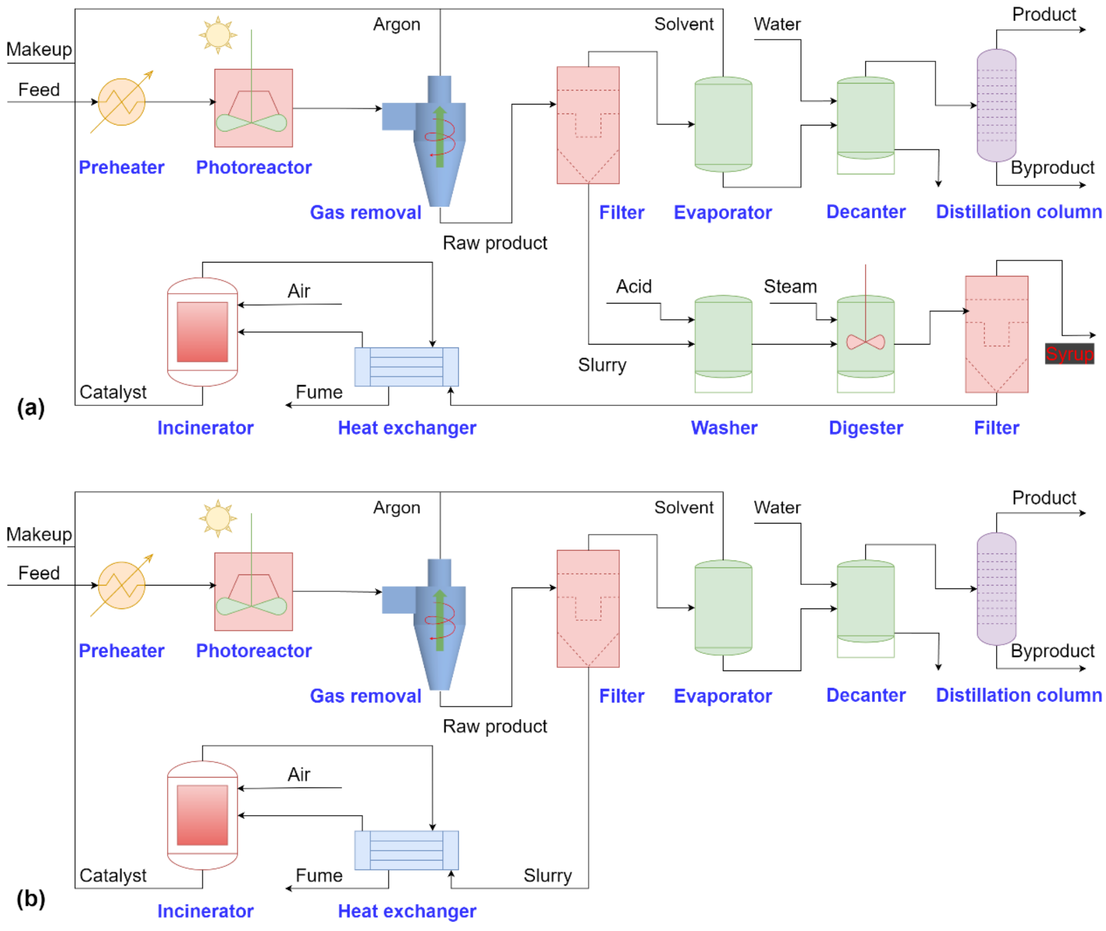

Fig. S16 | Process flow model of two scenarios. Scenario 1(a) and Scenario 2(b)

Table S9 | Key stream for process flow model

	Feed	Makeup	Raw product	Argon*	Solvent*	Slurry	Water	Product	Acid	Steam	Hydrolysis	Syrup	Catalyst
Mass	10	0.10	15.95	4.06	0.16	8.09	333.33	0.28	166.67	2833.33	3008.09	3001	1.67
Mass fraction/%													
Wheat straw	100												
Water		0.33	0.01	0.02	0.36		100		28.00	100	95.74	95.94	
Dioxane		35.98	0.63	0.56	25.05								
Ethanol		24.55	0.82	1.00	40.58								
Argon		39.15	25.07	98.40	1.06								
Lactic acid			7.01		23.19								
Acetic acid			0.05	0.02	2.07								
Levulinic acid			0.04		0.07								
Xylose			2.92		0.84								
Monomers			6.39		0.07			0.42					
Glucose											0.06	0.06	
Arabinose			4.53		1.30								
Cellulose			9.77			19.26							
4-			1.81		5.40			99.58					

Propylguaiacol						
Polysaccharide	12.08	23.81		0.06		
Slurry	18.43	36.34		0.10		
Catalyst	10.45	20.59				100
Sulfuric acid			72.00	3.99	4.00	

*1% of the flow was used as a purge gas to maintain the elemental balance of the system.

Techno-economic analysis (TEA)

The total production cost (TPC) of 4-propylguaiacol was estimated to evaluate the economic feasibility of this work. The assumed annual plant operating time is 8000 h and the total project life is taken to be 20 years. Depreciation cost is estimated with a straight-line depreciation method.

The total capital investment (TCI) consists of fixed capital investment (FCI) and working capital. The FCI of each equipment is estimated according to its inside battery limits (ISBL), outside battery limits (OSBL), and indirect costs (IDC). The ISBL was determined by the Aspen Process Economic Analyzer, while the OSBL and IDC were estimated to be ratios of the ISBL. Working capital is used to maintain plant operation, which was estimated at 10% of the FCI[29].

The TPC is estimated by raw materials cost, consumables cost, utilities cost, operating and maintenance cost, plant overhead cost, administrative cost, and byproduct revenue. The raw materials cost, consumables cost, utilities cost, and byproducts revenue were calculated by multiplying production inventories (shown in Table S9) with market prices (shown in Table S10). Other costs were estimated as coefficients of the FCI and operating labor costs. The operating labor cost was estimated according to operator salaries, plant scale, and equipment characteristics[29].

Table S10 | Key parameters for techno-economic analysis

**Parameter**		**Remarks**
TCI		(v)+(vi)
Inside battery limits (ISBL)	(i)	Installed equipment cost
Outside battery limits (OSBL)	(ii)	40% of (i)
Direct costs	(iii)	(i) + (ii)
Indirect costs (IDC)	(iv)	60% of (iii)
Fixed capital investment (FCI)	(v)	(iii) + (iv)
Working capital	(vi)	10% of (v)
TPC		(1)+(2)+(3)+(4)+(5)+(6)+(7)+(8)–(9)
Raw materials cost	(1)	Wheat straw: 50 CNY/t
Consumables cost	(2)	Hydrochloric acid: 82 CNY/t
		Sulfuric acid: 400 CNY/t
		Ethanol: 6000 CNY/t
		Dioxane: 5000 CNY/t
		Argon: 1000 CNY/t
Utilities cost	(3)	Steam: 200 CNY/t
		Process water: 15 CNY/t
Operating and maintenance cost	(4)	(4.1)+(4.2)+(4.3)+(4.4)+(4.5)
Operating labor	(4.1)	100000 CNY/operator year, 58 operators
Direct supervisory and clerical labor	(4.2)	20% of (4.1)
Maintenance and repairs	(4.3)	2% of (v)
Operating supplies	(4.4)	0.8% of (v)
Laboratory charge	(4.5)	15% of (4.1)
Depreciation cost	(5)	Lifetime: 20 years, salvage value: 4%
Plant overhead cost	(6)	60% of ((4.1)+(4.2)+(4.3))
Administrative cost	(7)	2% of TPC
Distribution and selling cost	(8)	2% of TPC

Byproduct revenue	(9)	Glucose: 2800 CNY/t dry

Techno-economic analysis results

The TPC of 4-propylguaiacol in this work was estimated to be 44354 CNY/t in Scenario 2, which is lower than that (187877 CNY/t) in Scenario 1. The high TPC of 4-propylguaiacol in Scenario 1 is attributable to huge steam input in the cellulose hydrolysis process, which implies the deprecated process of cellulose hydrolysis in this work. Apart from the cellulose hydrolysis process, the processes are parallel in Scenario 1 and 2. The main contribution accounts for consumables cost and depreciation cost. The reason might be that small scale (2800 t 4-propylguaiacol per annum) designed based on the biomass supply and product demand restrict the usage of advanced but costly facilities to recover consumables. The detailed breakdown of the TPC of 4-propylguaiacol in Scenario 2 is shown in Fig. S17.

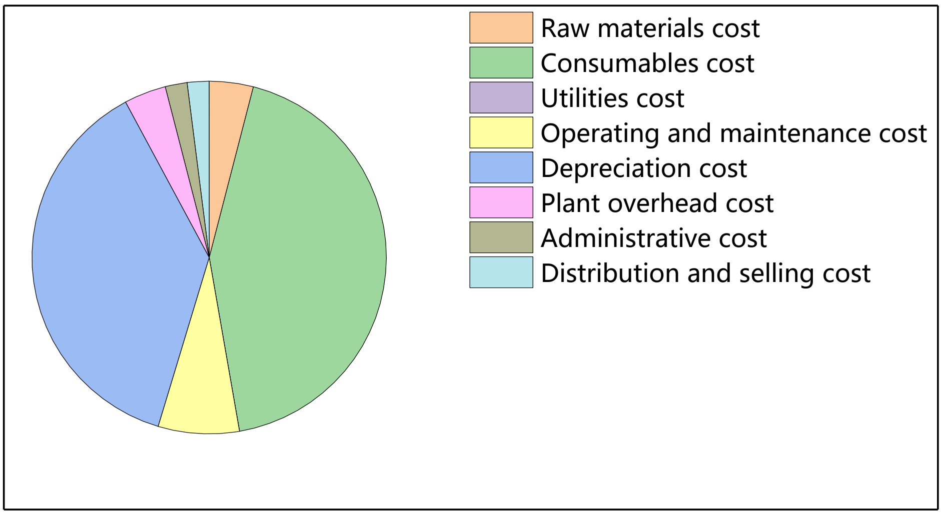

Fig. S17 | The detailed breakdown of total production cost (TPC) of 4-propylguaiacol in Scenario 2

Life cycle assessment (LCA)

Life cycle assessment (LCA) is used to evaluate greenhouse gas (GHG) emissions of this work. LCA is effective in evaluating environmental benefits, which is defined as the "compilation and evaluation of the inputs, outputs and potential environmental impacts of a product system throughout its life cycle".[30] The functional unit is defined as 1 metric ton of 4-propylguaiacol. The system boundary covers GHG emissions for the wheat straw collection, transport, production of consumables (hydrochloric acid, ethanol, dioxane, argon, and sulfuric acid), conversion process simulated above, and all upstream of required materials and utilities. The detailed system boundary of 4-propylguaiacol is depicted in Fig. S18.

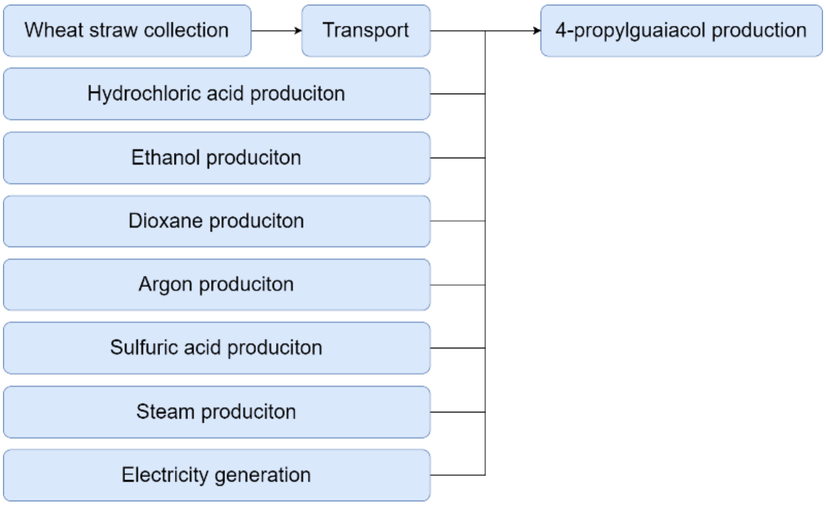

Fig. S18 | System boundary of this work

The GHGs involve CO_2, CH_4, and N_2O and were estimated in units of kg CO_2 equivalent according to a 100-year-time horizon[31], as Eq(S1).

$$GHG = E_{CO2} + 25E_{CH4} + 298E_{N2O} - 44/12C_{prod} \quad (S1)$$

where E_{CO2}, E_{CH4} and E_{N2O} represent emissions of CO_2, CH_4 and N_2O, respectively, kg; C_{prod} represents carbon sequestration in products, kg.

GREET 2020 software was taken to develop the model and link units[32]. The inventory of transformation from wheat straw to 4-propylguaiacol was shown in Supplementary Table S2 as mentioned above. Collection and transport of wheat straw, electricity, steam, hydrochloric acid, ethanol, dioxane, argon, sulfuric acid, and other

background inventory, were taken from the inherent database of GREET software. Life cycle GHG emissions were obtained by incorporating estimated material and energy into GREET 2020 finally.

Life cycle assessment results

The life cycle GHG emissions are 103.81 and 8.70 tCO_2eq/t 4-propylguaiacol in Scenario 1 and Scenario 2 (Fig. S16), respectively. The high GHG emissions are responsible for steam consumption (89.33%) in Scenario 1. The contribution of each factor to GHG emissions in Scenario 2 is shown in Fig. S19. It can be seen that the wheat straw accounts for the largest contribution, implying a higher conversion ratio is required.

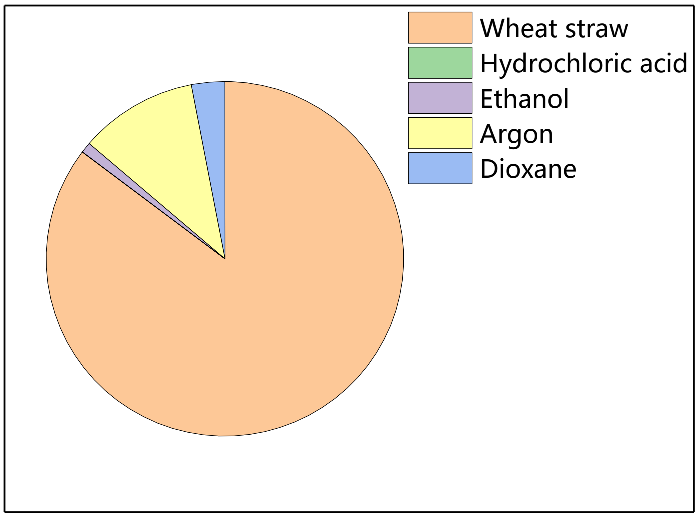

Fig. S19 | The detailed breakdown of life cycle GHG emissions of 4-propylguaiacol in Scenario 2

NMR data and spectra of lignin models

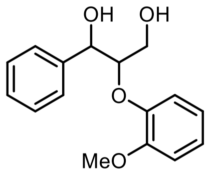

White solid. ^{1}H NMR (400 MHz, $CDCl_3$) δ 7.45 (d, *J* = 7.0 Hz, 1.4H), 7.41 – 7.27 (m, 3.6H), 7.07 (dd, *J* = 13.9, 8.0 Hz, 1.7H), 7.01 – 6.88 (m, 2.3H), 5.05 (d, *J* = 7.9 Hz, 1H), 4.19 (dd, *J* = 9.1, 4.7 Hz, 0.3H), 4.09 – 4.02 (m, 0.7H), 3.91 (s, 2H), 3.89 (s, 1H), 3.63 (dd, *J* = 12.6, 3.2 Hz, 1.2H), 3.49 (dd, *J* = 12.5, 3.9 Hz, 0.8H), 2.75 (s, 1H). ^{13}C NMR (101 MHz, $CDCl_3$) δ 151.73, 151.35, 147.57, 146.78, 139.78, 139.64, 128.56, 128.45, 128.24, 127.70, 126.04, 124.33, 121.71, 121.21, 121.15, 112.24, 112.19, 89.51, 87.48, 74.12, 72.93, 61.02, 60.58, 55.92.

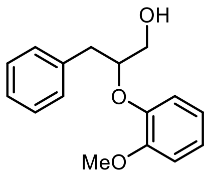

White solid. ^{1}H NMR (400 MHz, $CDCl_3$) δ 7.35 – 7.20 (m, 5H), 7.05 – 6.96 (m, 1H), 6.90 (dd, *J* = 8.1, 1.5 Hz, 1H), 6.84 (td, *J* = 7.7, 1.6 Hz, 1H), 6.76 (dd, *J* = 8.0, 1.6 Hz, 1H), 4.31 – 4.23 (m, 1H), 3.87 (s, 3H), 3.65 (ddd, *J* = 17.6, 12.1, 4.2 Hz, 2H), 3.15 (dd, *J* = 13.7, 6.6 Hz, 1H), 2.97 (dd, *J* = 13.7, 7.2 Hz, 1H), 2.83 (s, 1H). ^{13}C NMR (101 MHz, $CDCl_3$) δ 151.34, 147.49, 137.84, 129.55, 128.55, 126.55, 123.56, 121.41, 120.22, 112.16, 85.19, 77.03, 63.48, 55.87, 37.76.

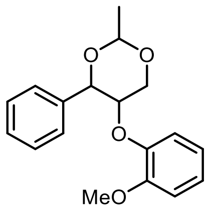

White solid. ^{1}H NMR (400 MHz, $CDCl_3$) δ 7.51 (d, *J* = 7.4 Hz, 2H), 7.33 (t, *J* = 7.4 Hz, 2H), 7.27 – 7.20 (m, 1H), 6.95 – 6.83 (m, 1H), 6.77 (dd, *J* = 8.1, 1.4 Hz, 1H), 6.71 – 6.60 (m, 1H), 6.46 (dd, *J* = 7.9, 1.5 Hz, 1H), 5.00 (q, *J* = 5.1 Hz, 1H), 4.93 (s, 1H), 4.44 (dd, *J* = 12.4, 1.4 Hz, 1H), 4.11 (d, *J* = 1.6 Hz, 1H), 3.93 (dd, *J* = 12.5, 1.3 Hz, 1H), 3.66 (s, 3H), 1.54 (d, *J* = 5.1 Hz, 3H). ^{13}C NMR (101 MHz, $CDCl_3$) δ 151.82, 147.51, 138.35, 127.96, 127.56, 126.78, 123.47, 121.55, 120.98, 113.20, 99.77, 80.50, 75.15, 68.84, 56.03, 21.15.

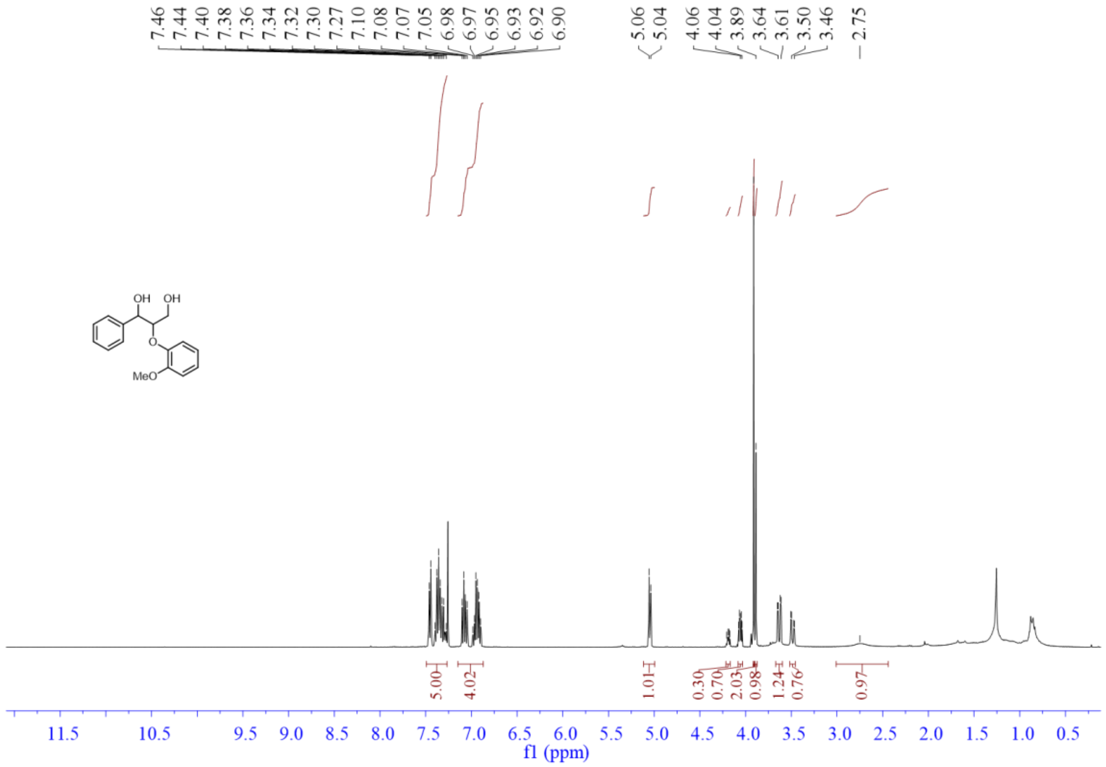
7.46 7.44 7.40 7.38 7.36 7.34 7.32 7.30 7.27 7.10 7.08 7.07 7.05 6.98 6.97 6.95 6.93 6.92 6.90
5.06 5.04 4.06 4.04 3.89 3.64 3.61 3.50 3.46 2.75
OH
OH
O
MeO
5.00
4.02
1.01
0.30
0.70
2.03
0.98
1.24
0.76
0.97
11.5 10.5 9.5 9.0 8.5 8.0 7.5 7.0 6.5 6.0 5.5 5.0 4.5 4.0 3.5 3.0 2.5 2.0 1.5 1.0 0.5
f1 (ppm)

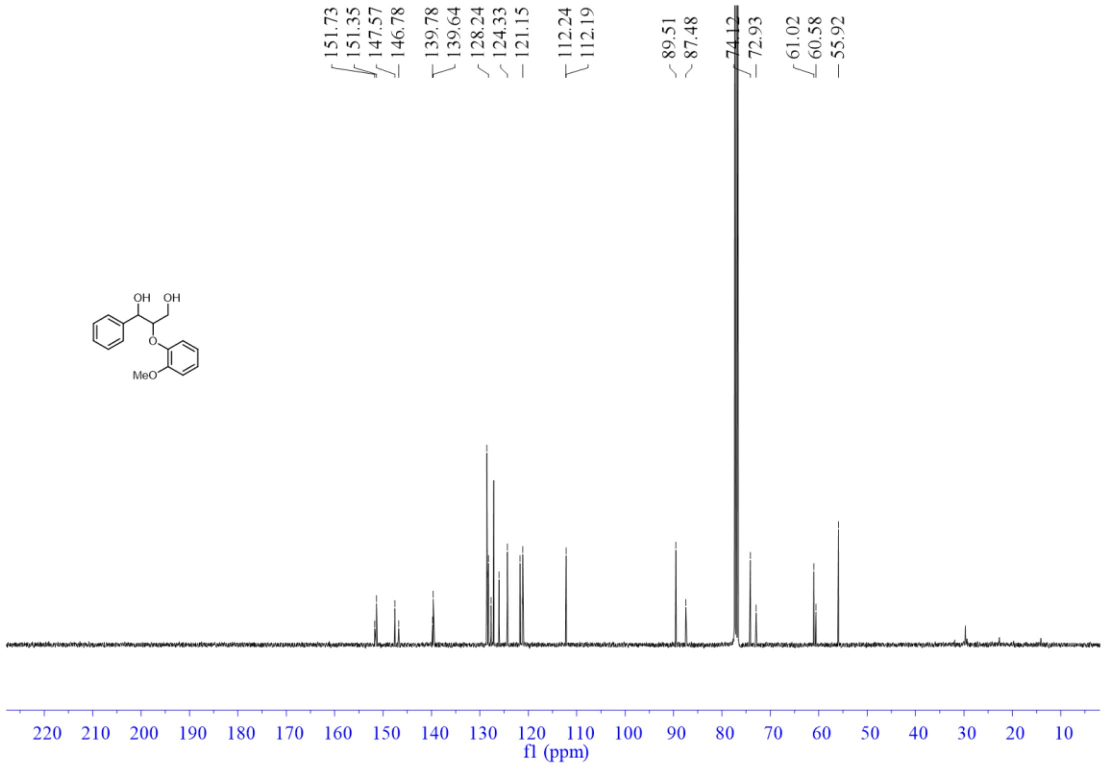
151.73 151.35 147.57 146.78 139.78 139.64 128.24 124.33 121.15 112.24 112.19
89.51 87.48 74.12 72.93 61.02 60.58 55.92
OH
OH
O
MeO
220 210 200 190 180 170 160 150 140 130 120 110 100 90 80 70 60 50 40 30 20 10
f1 (ppm)

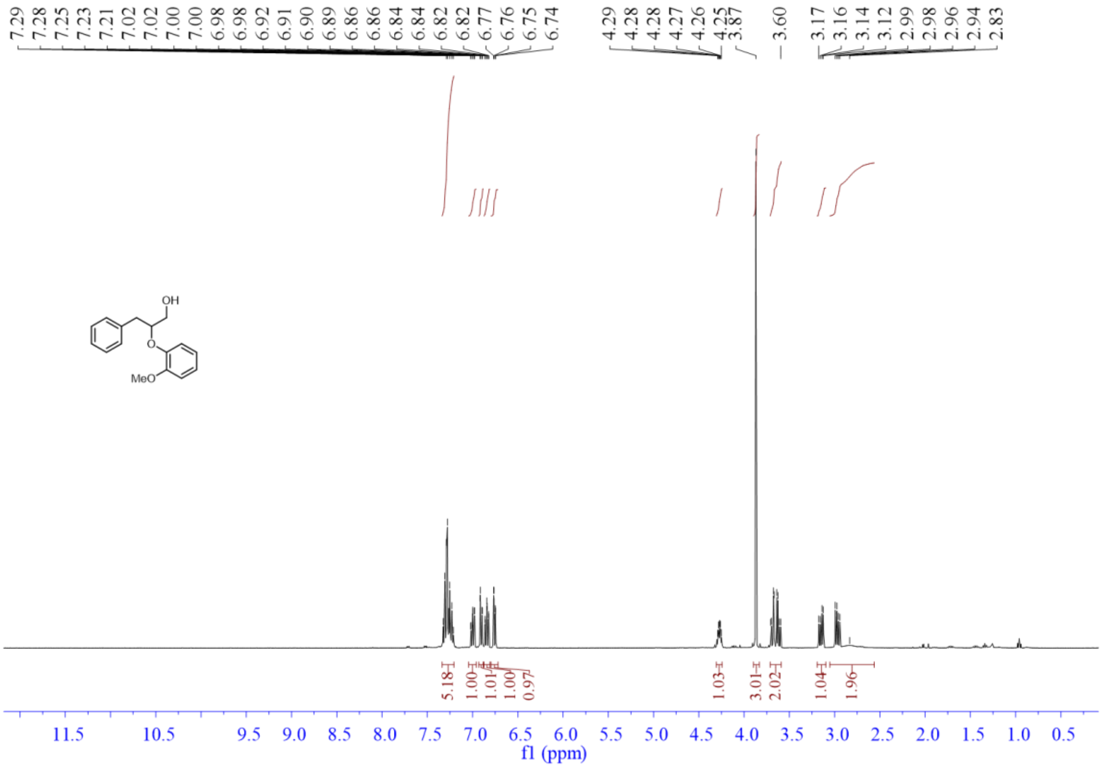

7.29 7.28 7.25 7.23 7.21 7.02 7.02 7.00 7.00 6.98 6.98 6.92 6.91 6.90 6.89 6.86 6.86 6.84 6.84 6.82 6.82 6.77 6.76 6.75 6.74
4.29 4.28 4.28 4.27 4.26 4.25 3.87 3.60
3.17 3.16 3.14 3.12 2.99 2.98 2.96 2.94 2.83
OH
O
MeO
5.18 1.00 1.01 1.00 0.97 1.03 3.01 2.02 1.04 1.96
f1 (ppm)

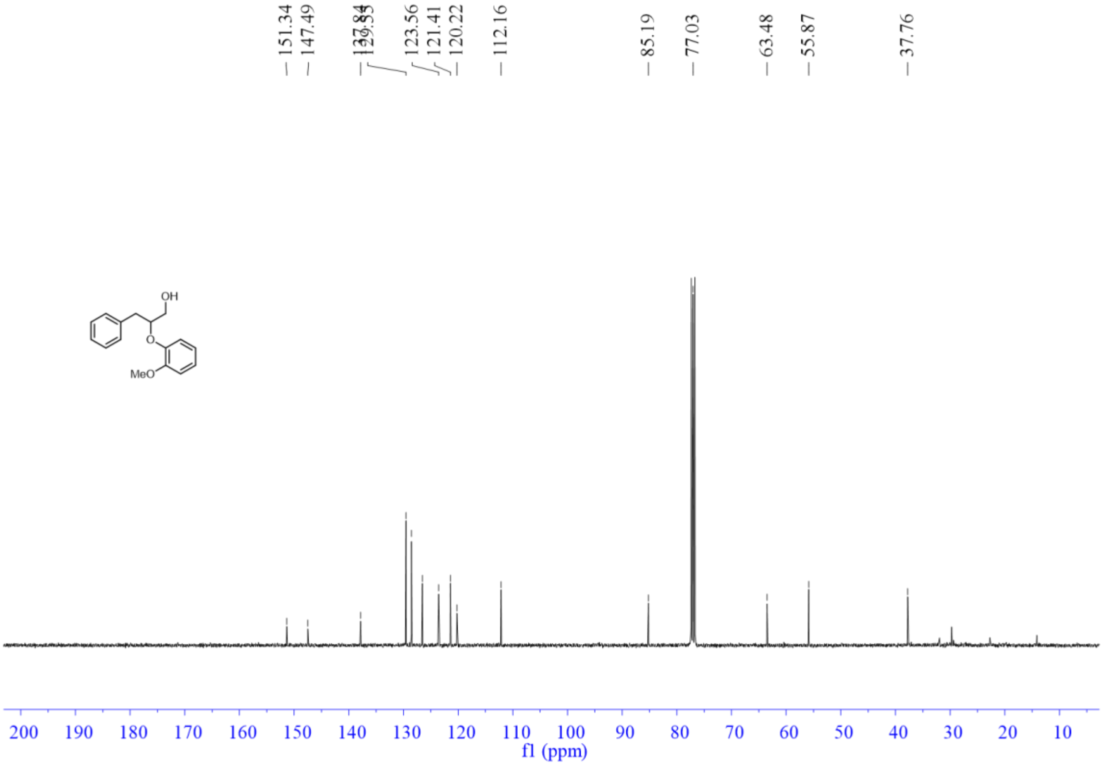

151.34 147.49 137.84 129.55 123.56 121.41 120.22 112.16 85.19 77.03 63.48 55.87 37.76
OH
O
MeO
f1 (ppm)

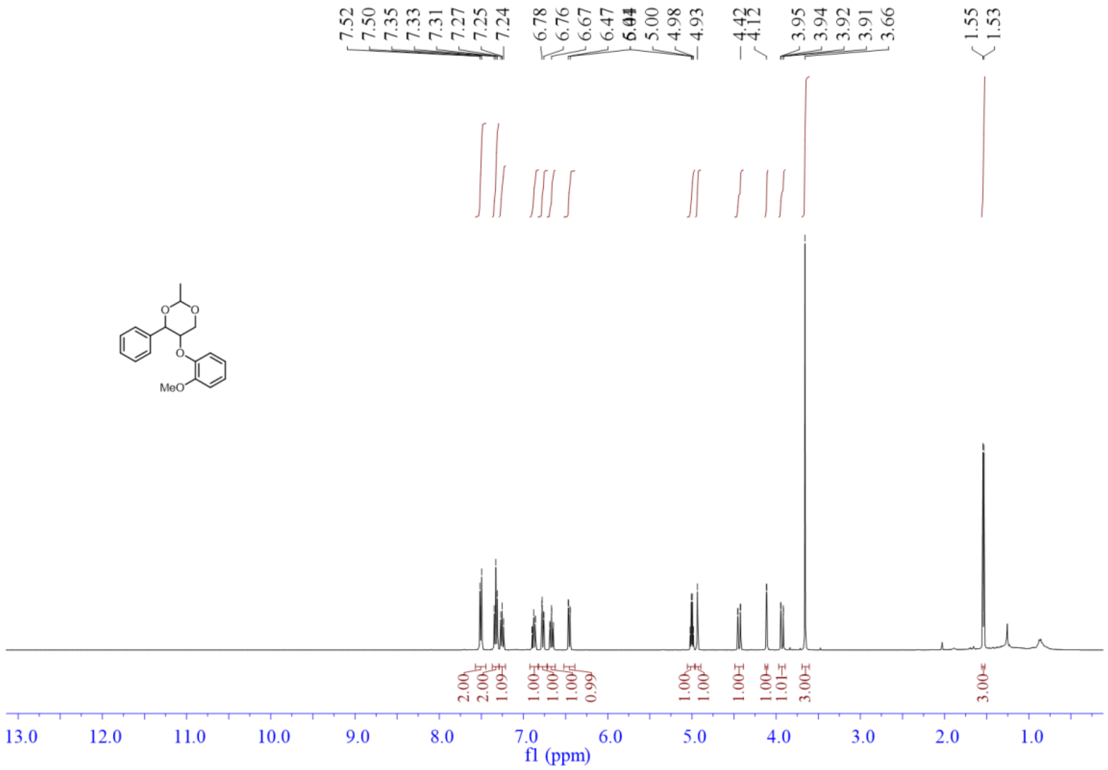
7.52
7.50
7.35
7.33
7.31
7.27
7.25
7.24
6.78
6.76
6.67
6.47
5.00
4.98
4.93
4.42
4.12
3.95
3.94
3.92
3.91
3.66
1.55
1.53
MeO
2.00
2.00
1.09
1.00
1.00
1.00
0.99
1.00
1.00
1.00
1.00
1.01
3.00
3.00
13.0 12.0 11.0 10.0 9.0 8.0 7.0 6.0 5.0 4.0 3.0 2.0 1.0
f1 (ppm)

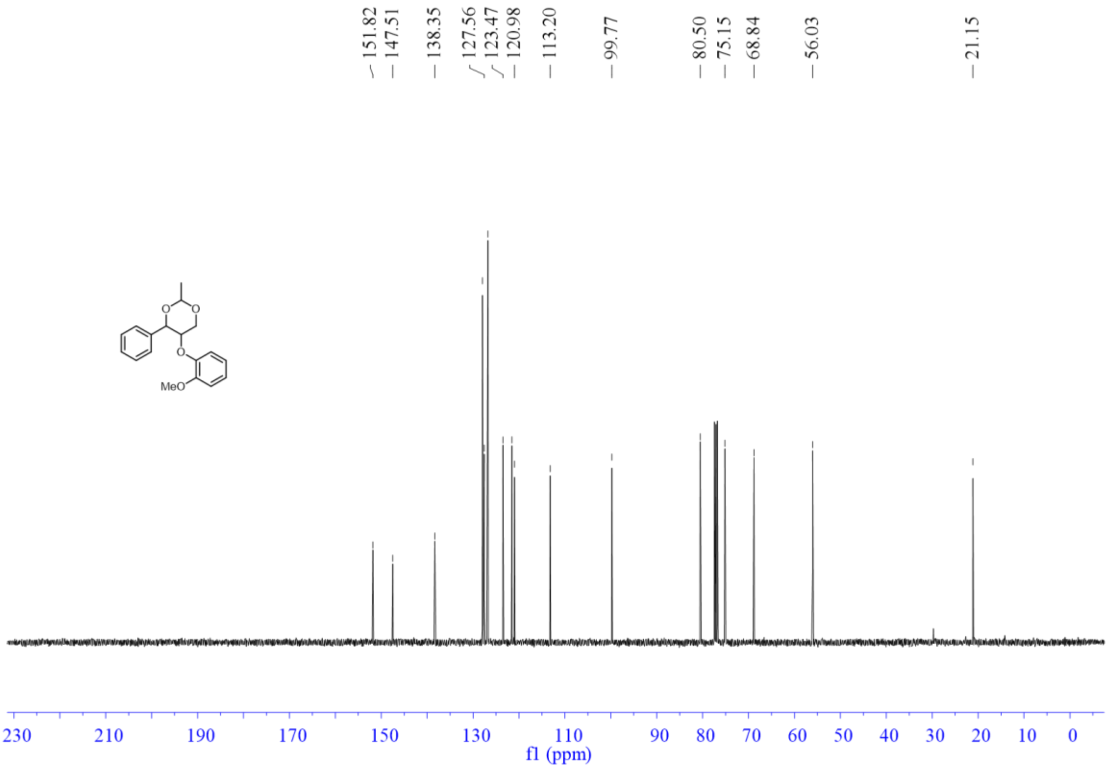
151.82
147.51
138.35
127.56
123.47
120.98
113.20
99.77
80.50
75.15
68.84
56.03
21.15
MeO
230 210 190 170 150 130 110 90 80 70 60 50 40 30 20 10 0
f1 (ppm)

Supplementary References

1 Li, H. *et al.* NH_2OH–Mediated Lignin Conversion to Isoxazole and Nitrile. ACS Sustainable Chemistry & Engineering 6, 3748-3753 (2018).

2 Li, H. *et al.* Photocatalytic Cleavage of Aryl Ether in Modified Lignin to Non-phenolic Aromatics. ACS Catalysis 9, 8843-8851 (2019).

3 Hu, J., Zhao, M., Jiang, B., Wu, S. & Lu, P. Catalytic Transfer Hydrogenolysis of Native Lignin to Monomeric Phenols over a Ni–Pd Bimetallic Catalyst. Energy & Fuels 34, 9754-9762 (2020).

4 Kenny, J. K. *et al.* Catalyst choice impacts aromatic monomer yields and selectivity in hydrogen-free reductive catalytic fractionation. Reaction Chemistry & Engineering 7, 2527-2533 (2022).

5 Facas, G. G., Brandner, D. G., Bussard, J. R., Román-Leshkov, Y. & Beckham, G. T. Interdependence of Solvent and Catalyst Selection on Low Pressure Hydrogen-Free Reductive Catalytic Fractionation. ACS Sustainable Chemistry & Engineering 11, 4517-4522 (2023).

6 Galkin, M. V. *et al.* Hydrogen-free catalytic fractionation of woody biomass. ChemSusChem 9, 3280-3287 (2016).

7 Song, Q. *et al.* Lignin depolymerization (LDP) in alcohol over nickel-based catalysts via a fragmentation–hydrogenolysis process. Energy & Environmental Science 6, 994-1007 (2013).

8 Muangmeesri, S. *et al.* Holistic Valorization of Hemp through Reductive Catalytic Fractionation. ACS Sustainable Chemistry & Engineering 9, 17207-17213 (2021).

9 Rautiainen, S. *et al.* Lignin Valorization by Cobalt-Catalyzed Fractionation of Lignocellulose to Yield Monophenolic Compounds. ChemSusChem 12, 404-408 (2018).

10 Liu, X. *et al.* Microwave-assisted catalytic depolymerization of lignin from birch sawdust to produce phenolic monomers utilizing a hydrogen-free strategy. Journal of Hazardous Materials 402, 123490 (2021).

11 Li, Y. *et al.* Hydrogen-Transfer Reductive Catalytic Fractionation of Lignocellulose: High Monomeric Yield with Switchable Selectivity. Angewandte Chemie International Edition 62 (2023).

12 Ren, T. *et al.* Highly selective reductive catalytic fractionation at atmospheric pressure without hydrogen. Green Chemistry 23, 1648-1657 (2021).

13 Dou, Z., Zhang, Z. & Wang, M. Self-hydrogen transfer hydrogenolysis of native lignin over Pd-PdO/TiO_2. Applied Catalysis B: Environmental 301, 120767 (2022).

14 Zhang, H., Zhang, H., Tian, S. & Fu, S. Deconstruction of biomass into lignin oil and platform chemicals over heteropoly acids with carbon-supported palladium as a hybrid catalyst under mild conditions. Bioresource Technology 341, 125848 (2021).

15 Zhou, H., Liu, X., Guo, Y. & Wang, Y. Self-Hydrogen Supplied Catalytic Fractionation of Raw Biomass into Lignin-Derived Phenolic Monomers and Cellulose-Rich Pulps. JACS Au 3, 1911-1917 (2023).

16 Liu, Y. *et al.* Rhodium-terpyridine catalyzed redox-neutral depolymerization of lignin in water. Green Chemistry 22, 33-38 (2020).
17 Oregui-Bengoechea, M. *et al.* High-Performance Magnetic Activated Carbon from Solid Waste from Lignin Conversion Processes. 2. Their Use as NiMo Catalyst Supports for Lignin Conversion. ACS Sustainable Chemistry & Engineering 5, 11226-11237 (2017).
18 Kong, X., Liu, C., Fan, Y., Li, M. & Xiao, R. Depolymerization of technical lignin to valuable platform aromatics in lower alcohol without added catalyst and external hydrogen. Fuel Processing Technology 242, 107637 (2023).
19 Jia, P., Wang, J. & Zhang, W. Catalytic hydrothermal liquefaction of lignin over carbon nanotube supported metal catalysts for production of monomeric phenols. Journal of the Energy Institute 94, 1-10 (2021).
20 Cheng, C., Li, P., Yu, W., Shen, D. & Gu, S. Catalytic hydrogenolysis of lignin in ethanol/isopropanol over an activated carbon supported nickel-copper catalyst. Bioresource Technology 319, 124238 (2021).
21 Lv, W. *et al.* Synergistic Effect of EtOAc/H_2O Biphasic Solvent and Ru/C Catalyst for Cornstalk Hydrolysis Residue Depolymerization. ACS Sustainable Chemistry & Engineering 5, 2981-2993 (2017).
22 Hu, J. *et al.* Catalytic transfer hydrogenolysis of lignin into monophenols over platinum-rhenium supported on titanium dioxide using isopropanol as in situ hydrogen source. Bioresource Technology 279, 228-233 (2019).
23 Zhang, B. *et al.* Cleavage of lignin C–O bonds over a heterogeneous rhenium catalyst through hydrogen transfer reactions. Green Chemistry 21, 5556-5564 (2019).
24 Zhang, J.-w., Lu, G.-p. & Cai, C. Self-hydrogen transfer hydrogenolysis of β-O-4 linkages in lignin catalyzed by MIL-100(Fe) supported Pd–Ni BMNPs. Green Chemistry 19, 4538-4543 (2017).
25 Jiang, L., Guo, H., Li, C., Zhou, P. & Zhang, Z. Selective cleavage of lignin and lignin model compounds without external hydrogen, catalyzed by heterogeneous nickel catalysts. Chemical Science 10, 4458-4468 (2019).
26 Wang, J. *et al.* Depolymerization of Native Lignin over Thiol Capped Ultrathin ZnIn2S4 Microbelts Mediated by Photogenerated Thiyl Radical. Angewandte Chemie International Edition 63 (2024).
27 Wu, X. J. *et al.* Solar energy-driven lignin-first approach to full utilization of lignocellulosic biomass under mild conditions. Nature Catalysis 1, 772-780 (2018).
28 Luo, N. *et al.* Visible-Light-Driven Self-Hydrogen Transfer Hydrogenolysis of Lignin Models and Extracts into Phenolic Products. ACS Catalysis 7, 4571-4580 (2017).
29 Zhao, Z., Jiang, J. & Wang, F. An economic analysis of twenty light olefin production pathways. Journal of Energy Chemistry 56, 193-202 (2021).
30 International Organization for Standardization. ISO14040 Environmental management - Life cycle assessment - Principle and framework. (2006).
31 Intergovernmental Panel on Climate Change. 2006 IPCC Guidelines for National Greenhouse Gas Inventories. (2006).

32 Argonne National Laboratory. The Greenhouse Gases, Regulated Emissions, and Energy Use in Transportation (GREET), url: https://greet.anl.gov/.
